# Exploring the Association of Angiotensin-(1–7) with Left Ventricular Mass Index in Youth with Hereditary Hypophosphatemia: A Pilot Study

**DOI:** 10.21203/rs.3.rs-10922460/v1

**Published:** 2026-09-20

**Authors:** Sivaram Kumareswaran, Jossiell Cortes, Andrew Michael South

**Affiliations:** The University of North Carolina at Chapel Hill; Noorda-COM: Noorda College of Osteopathic Medicine; Wake Forest School of Medicine: Wake Forest University School of Medicine

**Keywords:** Renin-angiotensin-aldosterone system, Angiotensin II, Angiotensin-Converting Enzyme 2, Cardiovascular-Kidney-Metabolic Syndrome, Pediatric Nephrology, Biomarkers, Left Ventricular Hypertrophy, Chronic Kidney Disease, X-linked Hypophosphatemic Rickets, Fibroblast Growth Factor 23

## Abstract

**Introduction:**

Fibroblast growth factor 23 (FGF23) regulates phosphate homeostasis and is associated with cardiovascular-kidney-metabolic syndrome in patients with chronic kidney disease in part mediated by angiotensin II. X-linked hypophosphatemia (XLH) is characterized by phosphate wasting due to excess FGF23 activity, but its associations with angiotensin peptides and cardiovascular disease remain unclear. We evaluated cross-sectional associations between angiotensin II, angiotensin-(1–7), and left ventricular mass index (LVMI) in youth with XLH.

**Methods:**

A pilot prospective cross-sectional study enrolled youth and young adults aged 2–24 years with XLH from nephrology and endocrinology clinics at one hospital. Individuals with acquired hypophosphatemia or inability to provide urine were excluded. LVMI was collected from echocardiograms while peptides were measured in blood and urine by radioimmunoassays. We assessed associations using Spearman correlation coefficients.

**Results:**

Among seven participants, 71% were female and median age was 12.8 years [IQR 7.7–13.7]. Seventy-one percent had a pathologic *PHEX* variant. Median peptide values were Ang II 56.0 pg/ml [47.2–58.1] and Ang-(1–7) 23.2 pg/ml [13.1–30.2] in blood and Ang II 32.1 pg/ml [27.5–103.2] and Ang-(1–7) 48.5 pg/ml [37.9–74.6] in urine. Median LVMI was 26.0 g/m^2.7^ [21.0–28.0] and 43.9 g/BSA [39.6–47.9]. Blood and urine angiotensin peptides were not correlated with either LVMI measure (*p* > 0.05).

**Discussion:**

In youth and young adults with XLH, blood and urine angiotensin peptide collection was feasible, but values were not associated with LVMI, suggesting ACE/Ang II and ACE2/Ang-(1–7) may not mediate FGF23-related cardiovascular-kidney-metabolic syndrome risk if confirmed. Larger studies are needed to determine if the renin-angiotensin-aldosterone system contributes to worse outcomes in FGF23-mediated XLH and should be targeted to improve cardiovascular-kidney-metabolic health.

## Introduction

Fibroblast growth factor 23 (FGF23) is a bone-derived hormone that regulates phosphate homeostasis. Elevations of FGF23 are associated with cardiovascular scarring and mortality [1]. FGF23 mediates a compensatory effect in patients with chronic kidney disease (CKD), due to retention of serum phosphorus in blood. This signaling triggers a pathological feed-forward loop with the renin-angiotensin-aldosterone system (RAAS) that induces left ventricular hypertrophy (LVH). This activation includes the suppression of angiotensin-converting enzyme 2 (ACE2), which cleaves angiotensin II (Ang II) resulting in Ang-(1–7), thus promoting inflammation and scarring of the heart muscle [2]. Genetic studies have identified missense mutations in *FGF23*, resulting in autosomal-dominant hypophosphatemia rickets (ADHR). Furthermore, ADHR, X-linked hypophosphatemia (XLH), and tumor-induced osteomalacia share common clinical and biochemical features characterized by hypophosphatemia resulting from renal phosphate wasting [3]. It remains unknown whether the FGF23-RAAS axis operates similarly in XLH, and whether patients with XLH carry elevated cardiovascular disease risk comparable to patients with CKD. In hypophosphatemic conditions such as XLH, the absence of confounding variables like uremia and tissue decay allows for a cleaner analysis of the relationship between Ang II/Ang-(1–7) and FGF23 [4].

Ang II and Ang (1–7) are thus primarily targeted in this study due to their opposing mechanisms in the RAAS cascade and their relationship with FGF23. Ang II acts via the Ang II type 1 receptor (AT_1_R) to promote vasoconstriction, inflammation, and fibrosis while Ang (1–7) acts through the Mas receptor to exert anti-hypertrophic effects [5]. In CKD, downregulation of ACE2 by overproduction of the FGF23 causes an overproduction of Ang II, accelerating cardiovascular damage [6]. FGF23’s relationship with the RAAS in patients with XLH could identify biomarkers for cardiovascular-kidney-metabolic syndrome risk in hypophosphatemic patients and could uncover targets within the RAAS-FGF23 signaling pathway. It is also unknown whether the alterations of Ang II and Ang (1–7) mirror the patterns that are observed in kidney failure or if there are specific mechanisms in hypophosphatemia disorders that cause a change in the interactions between FGF23 and the RAAS system. The objective of this study is to describe the cross-sectional relationships between Ang II and Ang-(1–7) and left ventricular mass index (LVMI) in patients with hereditary FGF23-related hypophosphatemia at a single hospital. The primary hypothesis is that higher Ang-(1–7) levels are associated with higher FGF23 levels, thus implying it confers a protective effect against FGF23’s deleterious actions on the heart. The secondary hypothesis is that among hereditary FGF23-related hypophosphatemia patients with LVH, Ang-(1–7) levels are lower relative to Ang II levels as compared to patients without LVH.

## Methods

### Study Design

This was a cross-sectional analysis of baseline data from a pilot prospective cohort study whose objectives were to demonstrate feasibility and provide preliminary data to inform the design including sample size calculations of future planned larger scale studies in this population with this ultrarare condition. The analytic plan goal was descriptive analysis. The study was approved by the Institutional Review Board at Wake Forest University School of Medicine (IRB00049606). We recruited youth and young adults who had hereditary FGF23-related hypophosphatemia and were receiving care from the Endocrinology and Nephrology clinics at Atrium Health Levine Children’s Brenner Children’s Hospital in Winston Salem, North Carolina over a two-year period from March 2018 – February 2020. Our hospital’s clinical protocol is that Endocrinology manages their bone disease, including prescribing burosumab, while Nephrology screens for and manages cardiovascular-kidney-metabolic syndrome. Our target enrollment was 30 participants based on anticipated patient censuses in the clinics over the proposed study time given this ultrarare condition via an initial i2b2 data query (Supplemental Table 1). We enrolled seven participants due to lower than anticipated eligible patients receiving care in the clinics during the study period. Recruitment was going to be extended past the original two-year end date given low enrollment numbers, but recruitment was first paused during the COVID-19 pandemic and then ended after the pandemic due to low anticipated enrollment. We obtained informed consent from parents or legal guardians and participants aged 18 years and older and informed assent from participants aged 7–17 years.

Inclusion criteria were a confirmed clinical diagnosis of hereditary FGF23-related hypophosphatemia and age between 2 and 24 years old to capture the pediatric to young adult spectrum of this ultrarare disease. Individuals with acquired FGF23-related hypophosphatemia were excluded from the study to avoid different pathophysiology that could confound the relationship between the exposure and the outcome. Individuals who were unable to provide a urine sample were also excluded from the study. All participants were planned to be followed longitudinally with study assessments at baseline, 6 months, and 12 months to assess the temporal relationships between these biomarkers and outcomes over time; due to attrition and subsequent small sample bias, we reported data from the baseline visit only. Study visits occurred in conjunction with clinic appointments to make it easier for participants to complete study procedures. Of the seven participants, all provided baseline blood and urine samples. For the six-month visit, only one participant was able to provide blood samples and two provided urine samples. For the 12-month visit, only three participants were able to provide blood samples and four provided urine samples. These missing data were due to missed clinic appointments before and during the pandemic (six participant-visits, one of which was rescheduled but the study team was unaware), a telehealth visit during the pandemic (one participant-visit), collected but insufficient blood and urine sample (one participant-visit), missing blood sample due to time constraints (one participant-visit), and no scheduled follow up in the clinics (one participant-visit).

### Data Collection

Demographic and clinical data, including age, sex assigned at birth, race, ethnicity, blood pressure, height, prescription for burosumab, echocardiogram results, and genetic study results were abstracted manually from the electronic health record.

### Laboratory Assessment

Blood (less than 5 mL) and urine samples were collected at each visit, along with routine clinical laboratory tests, to minimize additional blood draws. We measured Ang II and Ang-(1–7) in plasma and urine using radioimmunoassays validated to liquid chromatography-mass spectrometry in the CLIA-certified Biomarker Analytical Core laboratory at Wake Forest University School of Medicine [7]. No values were outside of the limits of detection for any laboratory test.

### Outcome Measures

All participants received a baseline echocardiogram to evaluate the heart’s structure and function per routine clinical practice in the Nephrology clinic for this patient population. Pediatric ultrasonographers and cardiologists at our hospital obtain and interpret all echocardiograms using a standardized protocol developed with Nephrology. We recorded left ventricular mass and mass indexed to both m^2.7^ and body surface area per national pediatric echocardiogram guidelines [8]. We recorded presence of LVH (defined as > 51 g/m^2.7^ for all participants, > 115 g/body surface area (BSA) in male participants, or > 95 g/BSA in female participants).

### Analysis Plan

Descriptive statistics were used to summarize the study variables and participants' characteristics. The continuous variables were reported as medians with interquartile ranges. Categorical variables were reported by using frequencies and percentages. Group comparisons were demonstrated through Fisher Exact test and Wilcoxon rank-sum test. We originally planned bivariate and multivariable generalized linear models to estimate beta coefficients for outcome measures on the continuous scale and OR for dichotomous or ordinal outcomes, but due to the low sample size we only used Pearson and Spearman correlation coefficients to assess continuous-variable correlations. We used Enterprise Guide, Version 7.1 of the SAS System for Windows (SAS Institute Inc., Cary, NC) for all analyses.

## Results

Of the seven participants enrolled in the study, 71% were female, 86% were white, no participant was Hispanic, and the median age at enrollment was 12.8 years [IQR 7.7 to 13.7] (Table 1). Seventy-one percent had a confirmed pathologic *PHEX* variant. The median systolic blood pressure was 110 mmHg [IQR 98 to 113] and median diastolic blood pressure was 61 mmHg [56 to 71]. Fifty-seven percent started burosumab for a median of 0.49 years prior to enrollment [IQR 0.38 to 1.13].

Eighty-six percent of participants provided measurable blood and urine samples. The median [IQR] peptide values were 56.0 pg/ml [47.2 to 58.1] for Ang II and 23.2 pg/ml [13.1 to 30.2] for Ang-(1–7) in the blood and 32.1 pg/ml [27.5 to 103.2] for Ang II and 48.5 pg/ml [37.9 to 74.6] for Ang-(1–7) in the urine. Fifty-seven percent had an echocardiogram a median of 1.16 years prior to enrollment [IQR 0.57 to 1.16]. The median [IQR] LVMI values were 26.0 g/m^2.7^ [21.0 to 28.0] and 43.9 g/BSA [39.6 to 47.9]. No participant had LVH.

In the blood, Ang II and Ang-(1–7) were not correlated with LVMI by g/m^2.7^ [Ang II ρ 0.21, *p* = 0.79; Ang-(1–7) ρ −0.63, *p* = 0.37] or g/BSA [Ang II ρ 0.8, *p* = 0.2; Ang-(1–7) ρ −0.4, *p* = 0.6] (Table 2). In the urine, Ang II and Ang-(1–7) were also not correlated with LVMI by g/m^2.7^ [Ang II ρ 0.32, *p* = 0.68; Ang-(1–7) ρ 0.95, *p* = 0.05] or g/BSA [Ang II ρ −0.2, *p* = 0.8; Ang-(1–7) ρ 0.8, *p* = 0.2].

## Discussion

In our pilot analysis of data from youth with hereditary FGF23-related hypophosphatemia enrolled in a single hospital, we were able to collect and analyze baseline samples for angiotensin peptides but experienced difficulties with longitudinal follow up. We did not observe a statistically significant association between angiotensin peptides and LVMI in our small sample size in this pilot study. If confirmed, this could suggest preservation of the ACE2/Ang (1–7) pathway of the RAAS pathway despite FGF23 activity and that the RAAS may not mediate FGF23-related cardiovascular-kidney-metabolic syndrome in this population earlier in life.

Studies have differing results from ours. Pi et al.’s study of wild-type rodents and Hyp mice demonstrated that Ang II administration increased FGF23 levels, induced LVH, and that FGF23 and Ang II expressed more deleterious cardiovascular effects [1]. FGF23 may suppress ACE2 expression, reducing cleavage of Ang II into Ang (1–7) and increasing Ang II’s deleterious cardiovascular effects. It is notable that our study population did not have an appreciable CKD.

Beck-Nielsen et al. and Brockman et al. both provide a review of the role of FGF23 in XLH’s pathophysiology [2, 9]. Elevated FGF23 is believed to contribute to cardiovascular abnormalities through multiple pathways. Cardiovascular findings in XLH patients were variably reported and usually confounded by conventional therapy, age, and genetic background [2, 9]. Our study is close to their framework in which we evaluate cardiovascular disease risk using an intermediate surrogate outcome, LVMI, within a hereditary FGF23 excess population. However, Beck-Nielsen et al. does not address Ang (1–7) or ACE2 and relies entirely on secondary literature review rather than original data collection [9].

Studies in CKD populations have shown that elevated FGF23 levels are associated with LVH and other adverse cardiovascular outcomes, mostly attributed to impairment of the ACE2/Ang (1–7) counter-regulatory pathway [10]. These studies usually involve adults with impaired kidney function and uremia, where FGF23 levels are more elevated than in patients with XLH [1, 10]. Our study differs from CKD literature in patient age, kidney function status, excess FGF23, and absence of uremia. This distinction highlights that the RAAS pathway in XLH may be fundamentally different than that observed in CKD [11]. Hernandez-Frías et al. conducted a similar study of pediatric XLH patients and found LVH present in their cohort but found no correlation between FGF23 levels and LVH [12]. This shows that FGF23 alone may be insufficient to contribute to cardiovascular-kidney-metabolic syndrome risk within this population [12].

Several alternate hypotheses should be considered and inform future study design. First, it may be that intact kidney function preserves appropriate ACE2 activity; thus, the kidney can cleave Ang II into Ang (1–7) regardless of FGF23 levels [13]. In CKD, kidney damage reduces the structural availability compared to XLH where kidney structure remains intact despite excess phosphate excretion [14]. It could also be possible that lower absolute levels of FGF23 in XLH compared to CKD may mean that FGF23 levels are insufficient to significantly suppress ACE2 expression in this population, potentially meaning there is a threshold that must be reached before FGF23 can impair ACE2/Ang (1–7) expression as seen in CKD. Another potential reason could be that there may be a compensatory upregulation of ACE2/Ang (1–7) as a response to elevated Ang II in these patients, causing a mechanism that is overwhelmed in CKD but remains functional in milder, hereditary FGF23 excess [15]. Understand which of these predominates would have major implications for therapeutic targeting.

The immediate next steps include larger, longitudinal studies in the same population of youth with excess FGF23 with XLH to determine whether the relationships observed in this population still hold the same. Ideally, such studies should begin diagnosis before treatment is initiated, which affects FGF23 levels and could affect RAAS expression. Future studies should also include an echocardiographic assessment at multiple time points to track changes in left ventricular structure and function to assess changes with elevated FGF23 and RAAS biomarker levels. Studies comparing patients with burosumab versus conventional phosphate and calcitriol therapy would be valuable, as they can determine if FGF23 reduction contributes to normalization of the Ang II/Ang (1–7) ratio and ultimately reduces longer term cardiovascular-kidney-metabolic syndrome risk [16]. Expanding older adult patients would also further clarify the preserved counter-regulatory pathway and see if it persists with age or eventually becomes impaired, potentially explaining variable cardiovascular findings in the adult XLH cohort [17]. Finally, mechanistic studies which target evaluation at ACE2 activity and expression in this population would help validate the pathways proposed by Pi et al. [1].

If, on the other hand, larger studies demonstrate associations with RAAS components and measures of cardiovascular-kidney-metabolic syndrome in this population over time, these findings could have meaningful implications across multiple levels of healthcare. Measurements of Ang (1–7) and the Ang II/Ang (1–7) ratio could become routine components of cardiovascular-kidney-metabolic health risk assessment [2, 6]. Clinicians managing XLH and related kidney disorders could use biomarkers in the RAAS system to easily identify patients at higher cardiovascular-kidney-metabolic syndrome risk and be sure to intervene earlier for more targeted management of XLH-related morbidities [12]. At the healthcare system level, these observations support a potential integration of cardiology comanagement into transdisciplinary care of patients with XLH [19]. From a public health and policy perspective, recognition of the effects of excess FGF23 in cardiovascular-kidney-metabolic syndrome risk could prompt updated clinical guidelines and screening recommendations, especially in patients with XLH, providing needed resources and support in this population with an ultrarare condition [3]. More broadly, if targeting the ACE2/Ang (1–7) pathway proves beneficial, it could open new therapeutic avenues for CKD patients as well, where cardiovascular mortality remains high, and current treatments are insufficient [10, 18].

This study does have limitations that must be acknowledged. The cross-sectional design and pilot status prevents us from establishing any sort of causality between RAAS biomarkers and LVMI, as we were not able to determine whether alterations in Ang (1–7) or the Ang II/Ang (1–7) ratio may cause changes in heart structure and function. Our sample is also limited to youth enrolled in a single center, which can limit the generalization to broader populations with XLH as variable cardiovascular findings across centers stated by Beck-Neilsen et al. suggests heterogeneity within this entire population [9]. The rarity of the disease also constrains sample size; this decreases statistical significance and increases the possibility of a type II error, a limitation that is also acknowledged in previous studies in cardiovascular disease outcomes of patients with XLH [17]. The current evidence base for cardiovascular-kidney-metabolic syndrome outcomes for patients with XLH relies mainly on small case reports, such as the two-patient report by Castellano-Martinez et al., with FGF23 reduction from burosumab treatment aiding in stabilizing LVH instead of reversal [13]. Though this is useful, it does not establish a generalization of cardiovascular-kidney-metabolic health in this population and FGF23’s potential effects, including via the RAAS [13].

We also did not directly measure ACE2 activity or expression; thus, the mechanistic link proposed between activated ACE2 function and evolved Ang-(1–7) levels remains ambiguous, and future studies should address this gap [1]. Conventional therapy with phosphate and burosumab may have confounded RAAS biomarker levels and cardiac outcomes in ways we were unable to account for fully, as conventional therapy has been shown to increase FGF23 levels and therefore carry cardiovascular disease risks [17]. This study’s strengths primarily lie on its focus on the pediatric population, which minimizes the confounding of age-related cardiovascular disease in measuring levels of RAAS biomarkers which prior studies had only theorized about [12], and majority of data contributed to these biomarkers are dominated by animal and CKD research, as well as rigorous methodology for measuring RAAS components [19].

In this pilot study of youth with FGF23-related hypophosphatemia from a single hospital, we demonstrated feasibility in measuring angiotensin peptides but difficulty in longitudinal follow up. We did not observe a statistically significant association between angiotensin peptides and LVMI in this small sample. If validated, these findings could challenge the current assumptions, derived mainly from CKD literature and animal models, that FGF23 suppresses the ACE2/Ang (1–7) counter-regulatory axis [12], which could suggest that the intact kidney function in younger patients with XLH may preserve this pathway [9]. Imagine a world where youth and young adults with XLH and related conditions can receive early cardiovascular-kidney-metabolic syndrome risk assessments based on accurate RAAS biomarker profiling and therapeutic targeting of the ACE2/Ang (1–7) pathway to reduces the burden of cardiovascular disease in not just this population, but for millions of other patients living with CKD worldwide [1, 3].

## Supplementary Material

This is a list of supplementary files associated with this preprint. Click to download.

• GAPSupplementalTable1.docx

## Figures and Tables

**Table 1:** Characteristics, Angiotensin Peptide Values, and Echocardiogram Outcome Measures of Participants with X-linked Hypophosphatemic Rickets

Age (yr)	Total Study Population *N* = 7
	12.8 [7.7, 13.7]
Female sex	5 (71%)
Systolic blood pressure (mmHg)	110 [98, 113]
Diastolic blood pressure (mmHg)	61 [56, 71]
Blood (*n = 6)*	
Angiotensin II (pg/ml)	56.0 [47.2, 58.1]
Angiotensin-(1–7) (pg/ml)	23.2 [13.1, 30.2]
Urine (*n = 6)*	
Angiotensin II (pg/ml)	32.1 [27.5, 103.2]
Angiotensin-(1–7) (pg/ml)	48.5 [37.9, 74.6]
LVMI (g/BSA), *n* = 4	43.9 [39.6, 47.9]
LVMI (g/m^2.7^), *n* = 4	26.0 [21.0, 28.0]

Median [IQR], *n* (%). BSA, body surface area.

**Table 2:** Spearman Correlation Coefficient Analysis between Angiotensin Peptide Values in Blood and Urine and Echocardiogram Outcome Measures of Participants with X-linked Hypophosphatemic Rickets

Blood (*n* = 4)	LVMI (g/m^2.7^)	LVMI (g/BSA)
	
Angiotensin II (pg/ml)	*ρ* 0.21, *p* = 0.79	*ρ* 0.8, *p* = 0.2
Angiotensin-(1–7) (pg/ml)	*ρ* −0.63, *p* = 0.37	*ρ* −0.4, *p* = 0.6
Urine (*n* = 4)		
Angiotensin II (pg/ml)	*ρ* 0.32, *p* = 0.68	*ρ* −0.2, *p* = 0.8
Angiotensin-(1–7) (pg/ml)	*ρ* 0.95, *p* = 0.05	*ρ* 0.8, *p* = 0.2

BSA, body surface area.

## References

[1] Pi M, Ye R, Han X, Armstrong B, Liu X, Chen Y, Sun Y, Quarles LD (2018) Cardiovascular interactions between fibroblast growth factor-23 and angiotensin II. Sci Rep 8(1):12398. 10.1038/s41598-018-30098-1 PMC6098163 30120363

[2] Böckmann I, Lischka J, Richter B, Deppe J, Rahn A, Fischer DC, Heineke J, Haffner D, Leifheit-Nestler M (2019) FGF23-mediated activation of local RAAS promotes cardiac hypertrophy and fibrosis. Int J Mol Sci 20(18):4634. 10.3390/ijms20184634 PMC6770314 31540546

[3] Liu P, Bai X, Wang H, Karaplis A, Goltzman D, Miao D (2009) Hypophosphatemia-mediated hypotension in transgenic mice overexpressing human FGF-23. Am J Physiology: Heart Circ Physiol 297(4):H1514–H1520 10.1152/ajpheart.00581.2009 19684183

[4] South A (2008) April–2022, August). FGF23 and Angiotensin-(1–7) in Hypophosphatemia [Clinical trial registration, NCT03489993]. ClinicalTrials.gov. https://classic.clinicaltrials.gov/ct2/show/study/NCT03489993

[5] Touyz RM, Montezano AC (2017) Angiotensin-(1–7) and vascular function. Hypertension 71(1):68–69. 10.1161/hypertensionaha.117.10406 29203630

[6] Zheng J, Hao H (2024) Targeting renal damage: The ACE2/Ang-(1–7)/mas axis in chronic kidney disease. Cell Signal 124:111413. 10.1016/j.cellsig.2024.111413. Epub 2024 Sep 16. 39293746

[7] South AM, Nixon PA, Chappell MC, Diz DI, Russell GB, Snively BM (2017) Antenatal corticosteroids and the renin-angiotensin-aldosterone system in adolescents born preterm. Pediatr Res 81(1):88–93 10.1038/pr.2016.179 PMC5646358 27636897

[8] Khoury PR, Mitsnefes M, Daniels SR, Kimball TR (2009) Age-specific reference intervals for indexed left ventricular mass in children. J Am Soc Echocardiogr 22(6):709–714 10.1016/j.echo.2009.03.003 19423289

[9] Beck-Nielsen SS, Mughal Z, Haffner D, Nilsson O, Levtchenko E, Ariceta G, de Lucas Collantes C, Schnabel D, Jandhyala R, Mäkitie O (2019) FGF23 and its role in X-linked hypophosphatemia-related morbidity. Orphanet J Rare Dis 14(1):58. 10.1186/s13023-019-1014-8 PMC6390548 30808384

[10] Dörr K, Kammer M, Reindl-Schwaighofer R, Lorenz M, Marculescu R, Poglitsch M, Beitzke D, Oberbauer R (2022) The effect of FGF23 on cardiac hypertrophy is not mediated by systemic renin-angiotensin-aldosterone system in hemodialysis. Front Med 9:878730. 10.3389/fmed.2022.878730 PMC9086596 35559350

[11] Leifheit-Nestler M, Große Siemer R, Flasbart K, Richter B, Kirchhoff F, Ziegler WH, Klintschar M, Becker JU, Erbersdobler A, Aufricht C, Seeman T, Fischer DC, Faul C, Haffner D (2016) Induction of cardiac FGF23/FGFR4 expression is associated with left ventricular hypertrophy in patients with chronic kidney disease. Nephrol Dialysis Transplantation 31(7):1088–1099. 10.1093/ndt/gfv421 PMC6388939 26681731

[12] Hernández-Frías O, Gil-Peña H, Pérez-Roldán JM, González-Sanchez S, Ariceta G, Chocrón S, Loza R, de la Cerda Ojeda F, Madariaga L, Vergara I, Fernández-Fernández M, Ferrando-Monleón S, Antón-Gamero M, Fernández-Maseda Á, Luis-Yanes MI, Santos F (2019) Risk of cardiovascular involvement in pediatric patients with X-linked hypophosphatemia. Pediatr Nephrol. ;34(6):1077–1086. 10.1007/s00467-018-4180-3.. Epub 2019 Jan 4. 30607568

[13] Castellano-Martinez A, Acuñas-Soto S, Roldan-Cano V, Rodriguez-Gonzalez M (2022) Left Ventricular Hypertrophy in Patients with X-Linked Hypophosphataemia. J Clin Res Pediatr Endocrinol 14(3):344–349. 10.4274/jcrpe.galenos.2021.2020.0287. Epub 2021 Mar 29. PMC9422913 33783172

[14] Maksimowski N, Williams VR, Scholey JW (2020) Kidney ACE2 expression: Implications for chronic kidney disease. PLoS ONE 15(10):e0241534. 10.1371/journal.pone.0241534 PMC7598523 33125431

[15] Rabelo L, Alenina N, Bader M (2011) ACE2–angiotensin-(1–7)–Mas axis and oxidative stress in cardiovascular disease. Hypertens Res 34(2):154–160. 10.1038/hr.2010.235 21124322

[16] Han X, Cai C, Xiao Z, Quarles LD (2020) FGF23 induced left ventricular hypertrophy mediated by FGFR4 signaling in the myocardium is attenuated by soluble Klotho in mice. J Mol Cell Cardiol 138:66–74. 10.1016/j.yjmcc.2019.11.149 PMC7195870 31758962

[17] Brener A, Cleper R, Baruch G, Rothschild E, Yackobovitch-Gavan M, Beer G, Zeitlin L, Kapusta L (2024) Cardiovascular health in pediatric patients with X-linked hypophosphatemia under two years of burosumab therapy. Front Endocrinol 15:1400273. 10.3389/fendo.2024.1400273 PMC11137213 38818505

[18] Jiang F, Yang J, Zhang Y, Dong M, Wang S, Zhang Q, Liu FF, Zhang K, Zhang C (2014) Angiotensin-converting enzyme 2 and angiotensin 1–7: Novel therapeutic targets. Nat Reviews Cardiol 11(7):413–426. 10.1038/nrcardio.2014.59 PMC7097196 24776703

[19] Bouzemane A, Vignot E, Derain Dubourg L, De Mul A, Molin A, Chapurlat R, Fontanges E, Delsart D, Akbari A, Huang SHS, McIntyre CW, Bacchetta J, Lemoine S (2024) Reassuring data on the cardiovascular risk in adults with X-linked hypophosphatemia receiving conventional therapy. J Clin Endocrinol Metabolism 109(2):e488–e494. 10.1210/clinem/dgad608 37843399

